# Kefir and Its Biological Activities

**DOI:** 10.3390/foods10061210

**Published:** 2021-05-27

**Authors:** Nor Farahin Azizi, Muganti Rajah Kumar, Swee Keong Yeap, Janna Ong Abdullah, Melati Khalid, Abdul Rahman Omar, Mohd. Azuraidi Osman, Sharifah Alawieyah Syed Mortadza, Noorjahan Banu Alitheen

**Affiliations:** 1 Department of Cell and Molecular Biology, Faculty of Biotechnology and Biomolecular Sciences, Universiti Putra Malaysia, Serdang 43400 UPM, Selangor Darul Ehsan, Malaysia; farahinazizi96@gmail.com (N.F.A.); drmuganti@gmail.com (M.R.K.); janna@upm.edu.my (J.O.A.); azuraidi@upm.edu.my (M.A.O.); 2 China-ASEAN College of Marine Sciences, Xiamen University Malaysia, Sepang 43900 UPM, Selangor Darul Ehsan, Malaysia; skyeap@xmu.edu.my; 3 Department of Biomedical Sciences, Faculty of Medicine and Health Sciences, Universiti Putra Malaysia, Serdang 43400 UPM, Selangor Darul Ehsan, Malaysia; melati@upm.edu.my; 4 Department of Veterinary Pathology and Microbiology, Faculty of Veterinary Medicine, Universiti Putra Malaysia, Serdang 43400 UPM, Selangor Darul Ehsan, Malaysia; aro@upm.edu.my; 5 Department of Biochemistry, Faculty of Biotechnology and Biomolecular Sciences, Universiti Putra Malaysia, Serdang 43400 UPM, Selangor Darul Ehsan, Malaysia; s_alawieyah@upm.edu.my; 6 UPM-MAKNA Cancer Research Laboratory, Institute of Bioscience, Universiti Putra Malaysia, Serdang 43400 UPM, Selangor Darul Ehsan, Malaysia

**Keywords:** kefir, bioactive compounds, probiotics, gut microbiota, therapeutic

## Abstract

Kefir is a fermented beverage with renowned probiotics that coexist in symbiotic association with other microorganisms in kefir grains. This beverage consumption is associated with a wide array of nutraceutical benefits, including anti-inflammatory, anti-oxidative, anti-cancer, anti-microbial, anti-diabetic, anti-hypertensive, and anti-hypercholesterolemic effects. Moreover, kefir can be adapted into different substrates which allow the production of new functional beverages to provide product diversification. Being safe and inexpensive, there is an immense global interest in kefir’s nutritional potential. Due to their promising benefits, kefir and kefir-like products have a great prospect for commercialization. This manuscript reviews the therapeutic aspects of kefir to date, and potential applications of kefir products in the health and food industries, along with the limitations. The literature reviewed here demonstrates that there is a growing demand for kefir as a functional food owing to a number of health-promoting properties.

## 1. Introduction

Kefir is a fermented drink with low alcohol content, acidic and bubbly from the fermentation carbonation of kefir grains with milk or water [1,2]. Its origin traces back to the Balkans, in Eastern Europe and in the Caucasus and, over time, its consumption has expanded to other parts of the world due to its health-giving properties [3]. This tart, viscous drink has become popular among people in countries such as the United States of America, Japan, France, and Brazil [4].

Kefir varies from other fermented products because of the specific property of its starter: The kefir grains. Kefir grains range in size from 1 to 4 cm in length and look like small cauliflower florets in shape (irregular and lobed-shaped) and color (from white to light yellow) [5]. This gelatinous and slimy structure is comprised of a natural matrix of exopolysaccharides (EPS) kefiran and proteins in which lactic acid bacteria (LAB), yeasts, and acetic acid bacteria (AAB) co-exist in symbiotic connection [2]. The most pre-dominantly found bacterial species in kefir grains are *Lactobacillus kefiranofaciens*, *Lacticaseibacillus paracasei* (basonym *Lactobacillus paracasei*), *Lactiplantibacillus plantarum* (basonym *Lactobacillus plantarum*), *Lactobacillus acidophilus*, and *Lactobacillus delbrueckii* subsp. *bulgaricus*. On the other hand, *Saccharomyces cerevisiae*, *S. unisporus*, *Candida kefyr*, and *Kluyveromyces marxianus* ssp. *marxianus* are the predominant yeast species present in kefir [3]. The microbiota of kefir grains may differ depending on the geographical origin of the kefir grains, which are strictly connected to the climate conditions [2]. In fact, the microflora composition in kefir may also differ depending on the substrate used in the fermentation process and culture maintenance method (fermentation time, temperature, degree of agitation, and ratio of kefir grains to substrate) [6]. It is recognized that this microbial diversity is responsible for the physicochemical features and biological activities of each kefir, although some major *Lactobacillus* species always exist because of their probiotic strain-specific properties [6,7].

In recent years, numerous studies on the putative health values of kefir as a natural beverage with probiotic microorganisms and functional organic substances have been reported. According to the Food and Agriculture Organization of the United Nations (FAO) and World Health Organization (WHO), probiotics refer to live microorganisms which, when applied in sufficient amounts, bestow a health benefit to the host. Additionally, evidence has shown that kefir’s exopolysaccharide, kefiran, has very significant physicochemical attributes and biological activities that certainly add value to the products [3,8,9,10]. Existing reports have suggested important health benefits from kefir beverage consumption, such as anti-microbial, anti-tumor, anti-carcinogenic, hypocholesterolemic effects, anti-hypertensive, anti-diabetic, immunomodulatory activity, and also improving lactose digestion [11]. All these health-promoting properties are linked to the kefir microorganisms, their interplays, and their metabolic products during the fermentation process [2].

This review reports the most current progress about kefir, its biological activities, and potential applications in the health and food industries.

## 2. Types of Kefir 

Generally, kefir may well be identified depending on the type of substrate used for fermentation, which are dairy and non-dairy kefir. The majority of the reported kefir studies has been emphasized on the advantages of kefir consumption that used milk substrates for fermentation compared with their non-dairy counterpart [3,5,12,13,14]. Despite its status as a natural probiotic, the intake of dairy kefir beverage not suitable for lactose intolerant, vegan and dairy-product allergic users [4]. Thus, an alternative method of reaping the health benefits of kefir is through its alteration to non-dairy substrates.

The dairy and non-dairy kefir grains are quite similar to each other’s in relations of their structure, related microorganisms and their metabolic products during the fermentation procedure [4]. However, the constitution and prevalence of microbial diversity of kefir grains and the concentration of end bioproducts may differ depending on the carbon and energy sources (substrate used) available for grain fermentation [15,16]. As an outcome of the diverse microbial constituents that can become proved within kefir grains, varying kefir products with different microbiological, physicochemical, nutritional, and sensorial characterizations of these kefir drinks may be obtained [17].

**Figure 1:**
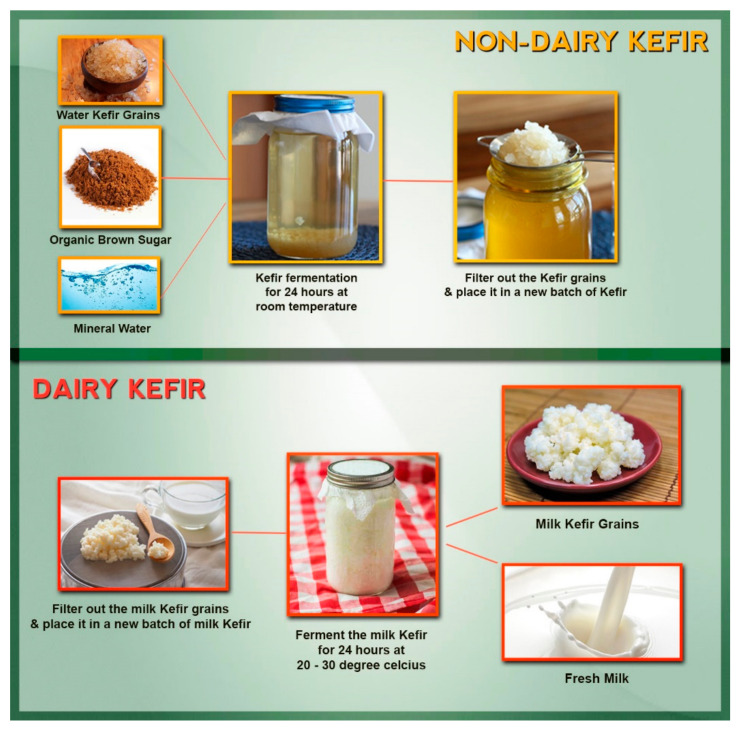
Different methods of fermenting non-dairy and dairy kefir.

Nevertheless, both dairy and non-dairy kefir are obtained by inoculating the starter culture, kefir grains, in the substrates at variable ratios (from 1 to 20% *w*/*v*) for 18–24 h at 20–25 °C (Figure 1). The fermentation procedure begins when bacteria and yeasts of the kefir grains find the proper culture requirements, which resulted in the increment of 5–7% grains’ biomass and the formation of various metabolites [18]. At the end of this procedure, kefir grains may split into new, smaller grains and liberate viable cells into the substrate, wherein the grains are isolated from kefir by sieving and re-use for the next inoculation [3].

### 2.1. Dairy Kefir

Since the 6th millennium BC, milk has featured in the human diet. In order to increase its shelf life, surplus milk was fermented [19]. Early humans discovered that preserved sour milk maintain their nutrients and is relatively more stable. Kefir is a homemade dairy beverage generated through fermentation of lactose in milk by bacteria and yeasts naturally existent in kefir grains. Traditionally, the fermentation of kefir was performed for 24 h at room temperature in goatskins, clay pots, or wooden buckets. The ruminants’ (cows, goats, sheep, camels, or buffalo) milk was applied as the fermentation substrate, in which at the end of this procedure kefir was isolated from the grains by drawing the beverage off [19].

Kefir can be produced either via the traditional method or a commercial process. The small-scale production calls for direct addition of kefir grains to milk that has been pasteurized and cooled to 20–25 °C. 

When producing at a large scale, kefir can be manufactured by using commercial starter cultures that are directly inoculated into the milk or by using the “Russian method”. This involves a backslopping procedure, a serial fermentation processes that begins with kefir produced from grains that are then used as natural starter cultures for milk fermentation [2,5]. Although kefir can be produced from different sources of animal milk, kefir made from cow’s milk remains the most popular in Eastern Europe [20]. Nevertheless, similar to other fermented milk products the condition of raw milk is crucial for kefir production. By and large, the raw milk for manufacturing kefir should be: rich inconstituent, low bacterial and somatic cell counts, and lack of pathogens or hindering substances, like antibiotics and disinfectant remains [5].

This yeast-lactic fermented milk produces a viscous and slightly effervescent beverage having a low alcohol content of nearly 0.08–2.0% [21]. Kefir is a non-curdled product and is described by a unique yeast-like flavor and a sparkling sense felt in the mouth. It owns this unique flavor to the symbiotic metabolic activities of several bacterial and yeast species, which include both proteolytic and lipolytic degradation of milk constituents. However, as a result of the complex community of microbiota in kefir grains, type of milk used, and type of manufacturing methods, the fermented kefir product has a highly diverse flavor, composition and metabolic products [19]. The typical composition of kefir contains 80–90% moisture, 0.2% lipid, 3.0% protein, 6.0% sugar, and 0.7% ash, and approximately 1.0% lactic acid and alcohol [22]. This fermentation process also produces metabolic products such as lactic and acetic acids, carbon dioxide, ethanol, acetaldehyde, acetoin, and other volatile compounds, minerals, essential amino acids, vitamins, folic acid, bacteriocins, bioactive peptides and some nutraceutical components [2].

### 2.2. Non-Dairy Kefir

Currently, kefir or kefir-like beverages are consumed throughout the globe, with information of kefir making in Ireland, Spain, Turkey, Malaysia, Indonesia, Tibet, and North and South America [19]. Water kefir, sugary kefir or tibico (tibico’s tepache) became very popular during the 20th century due to the health advantages associated with its intake [4]. 

Non-dairy kefir is a beverage made from the fermentation of kefir grains with a sugary solution, wherein the brown sugar solution is the main alternative substrate used for kefir fermentation [23]. Other non-dairy kefir prepared from fruit juices (apple, pineapple, grape, quince, kiwi, pear, pomegranate, melon, strawberry, tomato, coconut), vegetables (ginger, onion, soybean, fennel, carrot), and molasses (sugarcane, honey) are also suitable alternative substrates for the non-milk adaptation of kefir production [4,24,25,26,27,28,29]. These adaptations came about to allow non-dairy consuming and vegan individuals to reap the benefits of drinking kefir [30]. Regular daily intake of vegetables and fruits is strongly advocated for numerous positive health effects and disease prevention [31,32,33,34]. Thus, the production of fruit or vegetable juice-based fermented kefir beverage with may be perceived by consumers as healthy and provides an extra method to boost fruit and vegetable intake [35].

The non-dairy kefir fermentation is carried out by the kefir grains consisting of a consortium of yeasts mainly *Kluyveromyces*, *Candida* and *Saccharomyces*, and lactic acid bacteria (LAB), including the genera *Lactobacillus*, *Lactococcus*, *Leuconostoc*, and *Streptococcus*, embedded in a natural matrix of exopolysaccharides (EPS) kefiran [36,37,38]. Various species are found to have symbiotic associations and live or proliferate by sharing their bioproducts as energy supplies or growth-inducing factors, that may vary depending on non-dairy substrates used during kefir fermentation [39]. Indeed, sugary water or fruit juices contain water, sugar, and a mixture of nutrients; proteins, amino acids, vitamins and minerals that are suitable to prepare fermented beverages like kefir as they provide an ample medium for microbial expansion that could promote a fast rise of kefir grain biomass [40].

Non-dairy kefir beverages are traditionally produced by directly adding kefir grains to the pasteurized and cooled substrate and incubated for around 24 h at 25–30 °C. At the completion of fermentation, the grains are isolated from kefir by sieving, followed by washing, drying at room temperature and storage in a cooling tank for the next round of fermentation procedure [4]. The chemical composition and sensory feature of non-dairy kefir beverages vary corresponding to the substrate used, including sugars (sucrose, glucose, and fructose), organic acids (lactic, acetic, citric, tartaric, butyric, malic, and propionic acids), alcohols (ethanol, hexanol, and glycerol), and esters (ethyl propionate, ethyl hexanoate, octanoate, and decanoate). These metabolites afford distinctive flavor qualities for these products, such as revitalizing taste (due to presence of ethanol), fruity fragrance (due to presence of esters) and body and texture (due to presence of glycerol) that resulted in favorable consumer response for all analyzed products [4,35].

## 3. Biological Activities of Kefir 

The popular phrase “Let thy food be thy medicine and medicine be thy food,” by Hippocrates (400 BC) is used to highlight the idea of food to prevention or cure disease. Historically, kefir has been recommended for the remedy of several diseases, including tuberculosis, cancer, and gastrointestinal disorders when modern medical treatments were not obtainable [29]. In recent years, numerous studies on the bioactivities associated with kefir as a natural beverage have been reported. These putative health benefits could be ascribed both to the presence of probiotic microorganisms, as well as to the wide diversity of bioactive compounds yielded during the fermentation procedure [14].

### 3.1. Anti-Hypertensive 

Hypertension can lead to serious consequences such as heart attacks, strokes, and other cardiovascular diseases [41]. Data from the National Health and Morbidity Survey (NHMS) 2019, shows 3 out of 10 or 6.4 million people in Malaysia have hypertension and this risk increases with age. Shockingly, only half are aware that they have the disease and among these 90% are on medication but only 45% have their blood pressure under control (Institute for Public Health, 2020). Currently, kefir has raised attention in the scientific group due to its various beneficial effects on health, including anti-hypertensive effects, as well as being a safe and an economical homemade food [42,43,44,45]. The symbiotic metabolic events of a number of bacterial and yeast species in kefir, which include both proteolytic and lipolytic degradation of milk constituents create numerous biologically active peptides, including ACE-inhibitory peptides [43]. ACE-inhibitors block angiotensin-converting enzyme (ACE) from converting angiotensin I to potent vasoconstrictor angiotensin II. Consequently, it inhibits the production of aldosterone, a hormone that promotes the rise of serum sodium (Na) concentration, causing a surge in blood pressure and the breakdown of bradykinin, a hormone that has vasodilating action, influencing the decrease in blood pressure [14,42,46]. 

Various fermented kefirs with different strains of lactic acid bacteria have been reported for in vitro ACE inhibitory activity and in vivo anti-hypertensive effects [42,43,46,47,48]. In a current study conducted by Brasil et al. [42], the anti-hypertensive role of a soluble non-bacterial fraction of kefir on blood pressure and cardiac hypertrophy in hypertensive rats was stated to be facilitated by an increase in baroreflex sensitivity and decrease in angiotensin-converting enzyme activity. The result indicated that long-term treatment of the non-bacterial fraction of kefir promoted a significant decrease in both measurements of mean arterial pressure (MAP) and heart rate (HR) by improving baroreflex, and reducing cardiac hypertrophy in spontaneously hypertensive rats (SHRs), likely via ACE inhibition, and reduction of the TNF-α-to-IL10 ratio. The study on the impact of kefir on cardiac autonomic tones and baroflex sensitivity in spontaneously hypersensitive rats showed that daily chronic intake of a low dose of kefir had lowered the damage of the cardiac autonomic control of HR and baroflex sensitivity (BRS) in SHR [48]. In another study, the effects of kefir on endothelial cells and vascular responsiveness in spontaneously hypertensive rats (SHR) showed that kefir treatment for 60 days was able to improve the endothelial function in SHR by partially restoring the ROS/NO imbalance and the endothelial architecture due to endothelial progenitor cells recruitment [47]. These studies demonstrated the anti-hypertensive effect of both kefir’s bacterial, and non-bacterial fractions that could be ascribed to alterations in gut microbiota (postbiotic effect) that may vary depending on the bacterial strain or to other bioactive compounds produced by microbial action [42]. A study conducted by Aihara et al. [49] displayed that two tripeptides (Val-Pro-Pro and Ile-Pro-Pro) that are produced in the milk fermented with *Lactobacillus helveticus* have suppressing activities for angiotensin I-converting enzyme. Quirós et al. [46], found a potent angiotensin-converting enzyme (ACE)-inhibitory activity in commercial kefir manufactured by the fermentation of caprine milk and identified 16 peptides that were released from caseins. Of the 16, two (sequences PYVRYL and LVYPFTGPIPN) showed potent ACE-inhibitory properties. In another study conducted by Ebner et al. [43], 236 unique peptides were identified in kefir, and among these peptides, at least 12 had ACE inhibitory capacity. These studies suggest that kefir has the potential to be a coadjutant in the treatment of hypertension.

### 3.2. Anti-Cancer 

Cancer is the second leading cause of death globally, and the burden continues to grow in low- and middle-income countries to have access to timely quality diagnosis and treatment (World Health Organization, 2018). It is known that genetic factors play a large part in cancer risk. However, Weir et al. [50] ] reported that as much as 50% of cancers may be preventable through various lifestyle modifications, including practicing a healthy eating lifestyle. Therefore, the probiotics dietary aspects of kefir are vital as a potential coadjutant treatment or prevention in cancer. The anti-carcinogenic role of kefir and the fractions of kefir can be related to the prevention of cancer and retardation of tumor growth by apoptosis, immune response, modulation of intestinal microbiota, decreased tumor growth and DNA damage, anti-oxidative process, and inhibition of proliferation, and activation of pro-carcinogens [51]. Over the years, various in vitro and in vivo studies reporting the anti-cancer activities of kefir are shown in Table 1. The anti-carcinogenic effect of kefir and kefir fractions was studied for different cancer types, such as hematological cancers (leukemias and lymphoma), breast cancer, gastrointestinal system cancers (gastric and colorectal), and sarcoma (connective tissue tumor). 

In 2002, Liu et al. [52] performed an in vivo oral treatment of milk kefir and soymilk kefir in mice inoculated with sarcoma. They found that both types of kefir have resulted in significant suppression of tumor growth through stimulation of apoptotic cell lysis in tumors and a significant rise in IgA levels in mice, proposing that both kefir have anti-cancer attributes and have enhanced the mucosal resistance to gastrointestinal infection after 30 days of consumption. Another study has shown the immunoregulatory ability of kefir or kefir cell-free fraction (KF) on the immune response in mammary glands to delay tumor growth on a murine hormone-dependent breast cancer model. The results demonstrated the relationship between immune and endocrine systems as shown by the activation of immune cells (increased the number of IgA(+) cells, and decreased the Bcl-2(+) cells) and production of cytokines (increased IL-10, and decreased IL-6) [54,55]. Study in Syria demonstrated that several kefir namely alkaline kefir (AK), exopolysaccharides (EPS) and alkaline exopolysaccharides (AEPS) which were produced by a novel method at different concentrations exhibited anti-cancer properties against human sarcoma cells in vitro as reflected by the initiation of apoptosis. The novel method in this study was described as the new approach in the preparation of kefir being fermented in goat colostrum which involved extensive processes. The study showed that AK was superior for stimulation of apoptosis in sarcoma cells as compared to kefir and other kefir products [65]. Maalouf et al. [59] reported that a cell-free fraction of kefir exhibited its anti-proliferative effect and induced apoptosis by downregulating TGF-α and upregulating TGF-β1 mRNA expression on HTLV-1 negative malignant T-lymphocytes. A similar study by Rizk et al. [57] showed that kefir cell-free fraction caused the downregulation of TGF-α in HTLV-1 virus-infected human leukemia cell line. Another study by Rizk et al. [58] manifested that kefir treatment triggered an up-regulation of pro-apoptotic protein Bax and a down-regulation of anti-apoptotic protein Bcl-2 without altering p53 expression in both HTLV-1 negative/HTLV-1 positive cell lines. The apoptotic effect of *Lentilactobacillus kefiri* on human multidrug-resistant (MDR) myeloid leukemia (HL60/AR) cells in vitro was correlated with activation of caspase 3, decreased expression of Bcl-2, and decreased polarization of MMP [61]. Jalali et al. [64] indicated that kefir induced apoptosis and necrosis in the human acute erythroleukemia cell line (KG-1) in a dose- and time-dependent manner. 

The anti-carcinogenic outcome of kefir and kefir fractions had been demonstrated on gastrointestinal system cancers. Gao et al. [60], studied the anti-proliferative activity of cell-free fraction of Tibetan kefir on human gastric cancer cell SGC7901 in vitro. They found that SGC7901 cells treated with kefir were impeded in the G1/S phase, and both early and late apoptotic cells could be distinguished. It was also reported that the induction of apoptosis was effected via the up-regulation of bax and downregulation of bcl-2. In addition, Khoury et al. [62] showed the ability of kefir to inhibit proliferation and induce apoptosis in HT-29 and Caco-2 colorectal cancer cells. Another study on the effect of *Lentilactobacillus kefiri* on the apoptosis induction on gastric cancer cells (AGS) showed its association with the decreased polarization of mitochondrial membrane potential (MMP) and decreased Bcl2 expression, which suggested its therapeutic potential for the treatment of gastric cancers [63]. In a recent study, Riaz Rajoka et al. [10] characterized the isolated EPS produced by *Lentilactobacillus kefiri* MSR101 (MSR101 EPS) and studied their potential to block colon cancer (HT-29) cells growth. They found that MSR101 EPS illustrated anti-cancer activity against the HT-29 cancerous cell line and up-regulated the expression of Cyto-c, BAX, BAD, caspase3, caspase 8, and caspase 9. Moreover, the aqueous extract of kefir exhibited potent anti-oxidative activity in UVC-irradiated human melanoma HMV-1 cells and resulted in a remarkable decline in the intracellular reactive oxygen species (ROS) with enhanced DNA repair factors (thymine dimer repair-enhancing activity) [53]. The use of kefir supernatant as an adjuvant for doxorubicin (DOX) chemotherapy has also been studied owing to its chemo-sensitizing effects on multidrug-resistant (MDR) human colorectal cancer cells (HT-29) [66]. The results revealed that kefir and DOX enhanced the intracellular accumulation of ROS production in HT-29 MDR-developed cells and downregulation of ABC transporters. These outcomes were facilitated by the inactivation of ERK1/2 and NF-κB, and the activation of JNK. Such results indicate that kefir is potentially useful as a non-toxic adjuvant chemotherapy drug in multidrug-resistant developed cells. Altogether, these studies reveled the capability of kefir and kefir fraction as anti-cancer coadjutant in therapy.

### 3.3. Anti-Diabetic

A state of high blood glucose concentration or hyperglycemia, occurring from inadequacies in insulin secretion, action, or both is a complex chronic condition that positions patients at high risk for long-term macro- and microvascular complications [67]. According to the International Diabetes Federation (IDF) [68], 1 in 11 adults (20–79 years) has diabetes (463 million people), which make it a global pandemic. Without suitable treatment, persistent hyperglycemia may cause glucose toxicity, which may gradually damage the secretion of insulin. The requirement of absurdly high-cost insulin therapy is significant to reversing the toxic effect of high blood glucose levels on the pancreas [67]. However, in the last decade, growing evidence has shown the anti-diabetic effects of kefir as a potential low-cost therapeutic drug [67,69,70].

Earlier anti-diabetic effects of kefir can be observed in a study accomplished by Teruya et al. [71] in which they found water and methanol-soluble fractions of kefram-kefir were effective in the management of Type II diabetes by activating PI 3-kinase or other upstream molecules in the insulin signaling pathway, which resulted in the augmentation of glucose uptake. Later research on genetically diabetic mice (KKAy) fed kefiran for 30 days demonstrated a strong tendency for blood glucose levels to decrease as compared to the control group where the blood glucose concentrations increased continuously during the experiment [72]. In another study, Kwon et al. [73] showed that inhibition of hydrolytic enzymes called α-glucosidases and the pancreatic α-amylase can significantly decrease the postprandial increase of blood glucose levels after a mixed carbohydrate diet and, therefore, can be an important strategy in the management of type-II diabetes. By using this strategy, Kwon et al. [73] demonstrated that α-glucosidase inhibitory activity had increased moderately in kefir culture-mediated fermented soymilk supplemented with *Rhodiola* extracts exhibiting a better anti-diabetic functionality. A recent study also proved the capacity of kefir fermented soy milk (FSM) as manifested by inhibition of α-amylase activities at an IC50 value of 52.71 μg/mL [69]. They also found that the administration of FSM to hypercaloric high-fat-high-fructose diet (HFFD) rats inhibited intestinal and pancreas α-amylase activity by 26 and 31% as compared to untreated HFFD-rats, and consequently decreased the blood glucose by 36%. Hadisaputro et al. [74] studied the effects of plain kefir oral supplementation on the hyperglycemia of Wistar rats induced by streptozotocin for 30 days and revealed that kefir consumption was able to lower plasma glucose compared with the control group. Similar results were attained by Alsayadi et al. [70] who found that administration of kefir on streptozotocin-induced diabetic Wistar rats for 35 days showed lowered blood glucose levels in diabetic rats groups which were given water kefir instead of drinking water. Another study also supported the ability of kefir to the lower blood glucose level in STZ-diabetic rats to a significant level [75]. Different milk sources that were used for kefir fermentation also affect the anti-diabetic activity of kefir. The results obtained by Nurliyani et al. [76] on the anti-diabetic potential of kefir combination from goat milk and soy milk in rats induced with streptozotocin-nicotinamide, demonstrated that diabetic rats fed with the kefir combination had lower plasma glucose than rats fed with goat milk or soy milk kefir alone. A clinical trial on the effect of kefir on glucose and lipid profile control in 60 diabetic patients aged from 35 to 65 years which was conducted by Ostadrahimi et al. [77] showed that kefir decreased the fasting blood glucose and HbA1C levels and can be useful as a complementary or adjuvant therapy for the prevention of diabetes. Administration of kefir daily intake with metformin among the newly diagnosed type-2 diabetic adult male patients in the Gaza Government, has shown significant differences in some blood biochemical parameters [78]. The results demonstrated a decrease in fasting blood sugar (FBS), glycohemoglobin (HbA1c), and phosphorus with an increase in calcium among diabetic adult males upon kefir intervention. Furthermore, a study on the total incremental plasma glucose area under the curve (iAUC) for strawberry kefir, orange kefir, and low-fat plain kefir showed kefir as a low- to moderate-glycemic index (GI) food, which indicated its suitability for individuals with diabetes [79]. 

### 3.4. Anti-Microbial

According to Van Wyk [11] one of the aspects of the probiotic effect of kefir is the fact that the kefir microbiota produces anti-microbial metabolites. This anti-microbial capacity may be ascribed to the presence of hydrogen peroxide, peptides (bacteriocins), ethanol, carbon dioxide, diacetyl, and organic acids (lactic and acetic acids), which inhibit pathogens, particularly in the intestinal mucosa. Kefir and kefir-associated strains have shown a multitude of anti-microbial activities as shown in Table 2.

In general, kefir showed bacteriostatic effects on Gram-negative bacteria, but it is was more effective against Gram-positive bacteria [14]. Suriasih (2011) reported kefir’s ability to exhibit antimicrobial activity against Gram-negative bacteria, *Salmonella* Typhi and *Escherichia coli*. The surface layer protein from *Lactobacillus acidophilus* showed that the kefir consortium could suppress the adhesion and invasion of intestinal pathogen-induced apoptosis by regulating the mitochondrial apoptotic pathway [95]. In another experiment, isolated *Lentilactobacillus kefiri* B6 strain from kefir showed killing action against a Gram-positive bacteria, *Listeria monocytogenes* when in the existence of galactooligosaccharide (GOS) in vitro [105]. Likewise, a study by Sirirat and Jelena [114] showed the significant bacterial inhibition effect of the rice milk-kefir against *Staphylococcus aureus*, *Bacillus subtilis*, *Escherichia coli*, and *Pseudomonas fluorescens*. Other kefir-derived *Lactobacillus* species, such as *Lactobacillus kefiranofaciens* 8U, which was predominant in Brazilian milk kefir grains, showed antagonistic behavior against several pathogens, including *Pseudomonas aeruginosa*, *Listeria monocytogenes*, *Salmonella typhimurium*, *Staphylococcus aureu,* and *Enterococcus faecalis* [89]. Similarly, a study examining the isolated strains of *Lactococcus lactis* and *Lacticaseibacillus paracasei* (basonym *Lactobacillus paracasei*) from kefir has been shown to exhibit antimicrobial action against *Escherichia coli*, *Salmonella enterica*, *Staphylococcus aureus* and *Listeria monocytogenes*, however, further investigation on the range of their antimicrobial activities is needed [94]. Additionally, Jeong et al. [9], showed the isolated exopolysaccharide (EPS), EPS_DN1 from kefir-derived bacteria exerted bactericidal effects against *Salmonella enteritidis* and *Listeria monocytogenes*. An in vivo study on infected burn injuries on the dorsal skin surface of 56 rats has also shown kefir gels ability against the *Pseudomonas aeruginosa* ranged from 250 mg/mL minimum inhibitory concentration (MIC) to 250 mg/mL minimum bactericidal concentration (MBC) [106]. Additionally, it has been demonstrated that a combination of kefir microorganisms exerted protection against diarrhea and enterocolitis triggered by *Clostridium difficile* [87]. A later study on the mixture of kefir isolated two lactobacilli, one Lactococcus, and two yeasts demonstrated protection on epithelial cells in vitro against *Shigella invasion* [109]. Moreover, a recent study of the behavior of *Staphylococcus aureus* revealed that the use of a high kefir grain-to-milk ratio may minimize foodborne contamination during artisanal kefir manufacture [115].

LAB isolated from kefir produces bacteriocins which may be partially accountable for the anti-microbial action in a large range of bacterial strains [5]. Isolated bacteriocin F1 from *Lacticaseibacillus paracasei* subsp. *paracasei* (basonym *Lactobacillus paracasei* subsp. *paracasei*) of Tibetan kefir has shown to inhibit both bacteria and fungi, such as *Escherichia coli*, *Staphylococcus aureus*, *Bacillus thuringiensis*, *Salmonella enterica*, *Shigella dysenteriae*, *Aspergillus flavus*, *Aspergillus niger*, *Rhizopus nigricans*, and *Penicillium glaucum* [86]. Ebner et al. [43] identified two antimicrobial peptides, caseicin B (VLNENLLR sequence) and casecidin 17 (YQEPVLGPVRGPFPIIV sequence), which showed inactivating activity against *Escherichia coli*. Dallas et al. [44] identified 25 peptides from peptidomic analysis of kefir microorganisms on bovine milk proteins, including six peptides (Casecidin-15, Casecidin-17, Caseicin B, Caseicin C, Isracidin, αs1-Casein f.30–37) with anti-microbial function. Moreover, the non-microbial fraction of kefir produced organic acid, lactate upon fermentation, and exerted a protective effect against intestinal pathogens (*Escherichia coli*, *Salmonella* spp. and *Bacillus cereus*) [116]. The results demonstrated a concentration-dependence of lactate on pathogen growth inhibition and invasion of epithelial cells. Such results indicate the significance of microbial metabolites in the anti-microbial activity of kefir. Thus, anti-microbial activities of kefir may be useful as a safe alternative for use in preservation of food products and reduction in foodborne pathogens during food production, and storage.

Interestingly, a recent review by Hamida et al. [117] proposed the potential of kefir and its by-products as protective agents against virus, such as Severe Acute Respiratory Syndrome Coronavirus 2′ (SARS-CoV-2) that caused Coronavirus disease 2019 (COVID-19), owing to its proven antiviral mechanism against viral infections (Zika, hepatitis C, influenza, rotaviruses). Kefir and its probiotic contents were shown to regulate the immune system to overcome infections from these viruses by stimulating immune-system responses and also by suppressing the pursuit of pro-inflammatory cytokines such as IL-1β, tumor necrosis factor (TNF)-α and IL-6. Consequently, the mode of action of kefir and its probiotic contents against these viruses highlights the possible efficacy against SARS-CoV-2.

### 3.5. Anti-Inflammation

Worldwide, the complications of neuroinflammatory diseases and inflammation in chronic disorders constitute to the primary cause of morbidity and mortality. Over the past years, growing evidence from both in vitro and in vivo studies displayed conclusive anti-inflammatory and immunomodulatory potentials, where kefir treatment proved to elevate the anti-inflammatory mediators while downregulating the pro-inflammatory cytokines. Vinderola et al. [118] studied the immunomodulatory effect of the exopolysaccharide produced by *Lactobacillus kefiranofaciens* in the intestinal mucosa level in mice by examining the cytokines and immunoglobulins profiles. The oral administration of exopolysaccharide in mice resulted in the gut mucosal response through elevating the production of IgA at both the small and large intestines, and inducing systemic immunity through the production of cytokines in the intestinal fluid and blood serum. Likewise, Carasi et al. [119] investigated the immunomodulatory properties of *Lentilactobacillus kefiri* isolated from kefir and discovered that its administration induced alterations in the gut microbiota composition. The study found that the *Lactobacillus kefiri* induced pro/anti-inflammatory cytokines production at different ratios. The study also established that the administration of *Lentilactobacillus kefiri* CIDCA 8348 to mice had elevated anti-inflammatory molecules, such as the IL-10, CXCL-1, and mucin 6 genes, and downregulated the expression of pro-inflammatory mediators, such as IFN-γ, GM-CSF, and IL-1β, in inductive and effector sites of the gut immune system. Rosa et al. [120] examined the effects of kefir supplementation on Spontaneously Hypertensive Rats (SHR) by analyzing the metabolic parameters, expression of oxidation and inflammatory markers, and glycemic index control. The study reported that kefir supplementation on SHR for ten weeks increased the expression of anti-inflammatory cytokine (IL-10), lowered the expression of pro-inflammatory cytokine (IL-1β), and decreased the products of lipid oxidation when compared to the positive control group.

The use of kefir peptides in several in vivo studies has also demonstrated its potent immunostimulating effects. Chen et al. [121] investigated the anti-inflammatory effects of kefir peptides produced from the fermentation of kefir grain with milk proteins on particular matter <4 μm (PM4.0) induced lung inflammation in NF-κB-luciferase+/+ transgenic mice. The study showed that the kefir peptides administration at both doses (150 mg/kg and 500 mg/kg) reduced PM4.0-induced inflammatory cell infiltration and inflammatory mediators’ expression such as TNF-α, IL-lβ, and IL-4 in lung tissue by inactivating NF-κB signaling. Following the current finding in 2020, Chen et al. [122] further studied the effects of the kefir peptides against oxidative stress and inflammation and their protective ability against renal dysfunction on aged salt-induced stroke-prone spontaneously hypertensive (SHRSP) rats. The study revealed a decrease in renal infiltration of inflammatory cells; reactive oxygen species (ROS) levels; histopathological lesions; and the release of vascular cell adhesion molecule-1 (VCAM-1), monocyte chemoattractant protein-1 (MCP-1), endothelin-1 (ET-1), and the cytokine nucleotide-binding oligomerization domain (NOD)-like receptor family pyrin domain containing 3 (NLRP3) and transforming growth factor-β (TGF-β) in salt-induced SHRSP rats. Additionally, the administration of kefir peptide to the SHRSP rats resulted in a significant increase in the glomerular filtration rate and the renal superoxide dismutase activity proving its potent protection against salt-induced chronic kidney disease. Investigation on the anti-inflammatory effects of kefir peptides on a rat model of adjuvant-induced arthritis also revealed lower arthritis score, lower histological severity score of arthritis in hind paws, and decreased severity of the bone erosion of the ankle joint indicating anti-inflammatory effects of the kefir peptides.

Interestingly, Hadisaputro et al. [74] explored the effects of plain kefir treatment on the glycemic status and immune responses of Streptozotocin-induced hyperglycemia Wistar rats. The study showed that the plain kefir supplementation significantly reduced the blood glucose, the level of pro-inflammatory cytokines (IL1 and IL6), the level of TNF, and enhanced the level of anti-inflammatory cytokine (IL10), suggesting a decrease in the subsequent effect of free radicals and lipid peroxidation. Additionally, a study by Seo et al. [123] found that kefir produced extracellular vesicles (EV) that inhibit the inflammatory cytokine production by mitigating TNF-induced inflammation in intestinal cells. The study showed that treatment of each kefir-derived *Lactobacillus* EV (K-LEV) on TNF-α-stimulated Caco-2 cells had significantly reduced the mRNA expression and IL-8 secretion. Western blot analyses of the study revealed that such effect was due to TNF-α signaling inhibition mediated by reducing p65 phosphorylation, which is a subunit of NF-kB. The study also showed that treatment with K-LEV in inflammatory bowel disease mice had significantly decreased the disease-related symptoms, such as body weight loss and rectal bleeding, and improved stool consistency. Similarly, Santanna et al. [124] investigated the effects of a soluble, nonbacterial fraction of kefir on the progression of atherosclerosis in low-density lipoprotein receptor-deficient (LDLr−/−) mice. The soluble, nonbacterial kefir supplementation to LDLr−/− mice has led to a 50% reduction of pro-inflammatory cytokine (IL-6), 42% of reduction of TNF-α/IL-10, and a 74% increase in the anti-inflammatory cytokine (IL-10) level. Furthermore, Lee et al. [125] studied the anti-inflammatory and anti-allergic effects of kefir in an ovalbumin-induced mice asthma model. The study revealed that an hour of kefir pre-treatment before the ovalbumin challenge had significantly suppressed the ovalbumin-induced airway hyper-responsiveness, and significantly inhibited the increase in total inflammatory cell and eosinophil counts induced by ovalbumin and bronchoalveolar lavage fluid, respectively. In bronchoalveolar lavage fluid, the levels of interleukin-4 and -13, and total immunoglobulin E were reduced to normal levels. 

Kang et al. [126] investigated the anti-inflammatory effects of *Lactobacillus kefiranofaciens* subsp. *kefirgranum* PRCC-1301-derived extracellular vesicles (PRCC-1301 EVs) on intestinal inflammation and intestinal barrier function. The PRCC-1301 EVs inhibited the expression of the pro-inflammatory cytokines in Caco-2 cells and elevated intestinal barrier function by conserving intestinal cell integrity and the tight junction. In acute and chronic murine colitis models, the PRCC-1301 EVs mitigated body weight loss, histological damage, colon shortening, and the reduction of phosphorylated NF-κB p65 and IκBα in colon tissue sections, indicating the potent anti-inflammatory effect of PRCC-1301 EVs in inhibiting the NF-κB pathway and improving intestinal barrier function. Ali et al. [127] studied the protective role of kefir in attenuating γ radiation-induced hepatotoxicity. The results from this study demonstrated that kefir treatment had significantly decreased the γ radiation-induced hepatic function impairment, hepatic histological alterations, and dyslipidemia. The study indicated that the kefir inhibits the induced inflammation and improves the state of oxidative stress. A recent study by Tung et al. [128] reported the effects of kefir peptides on high-fat diet-induced atherosclerosis in apolipoprotein E knockout mice. The kefir peptides treatment has significantly improved the development of atherosclerotic lesion by attenuating oxidative stress, macrophage accumulation, endothelial dysfunction, aortic lipid deposition, and inflammatory immune response. Another study by Yin et al. [129] revealed the anti-inflammatory effects of Micro Integral Membrane Protein (MIMP) of *Lactiplantibacillus plantarum* (basonym *Lactobacillus plantarum*) on dextran sodium sulfate (DSS)-induced colitis mice model. The study found that the MIMP treated group had a significant decrease in the inflammation scores, reduced expression of pro-inflammatory cytokines such as IFN-γ, IL-17, and IL-23, and increased expression of anti-inflammatory cytokines, such as IL-4 and IL-10, compared to those in the DSS-treated group. The study also found that the gut microbiota dysbiosis caused by DSS-induced inflammation was improved by the MIMP treatment. Additionally, the MIMP treatment in the co-culture model of PBMCs and CaCO-2 resulted in suppression of lipopolysaccharide-induced inflammation, modulation in the expression of inflammatory cytokine through the toll-like receptor 4 pathway, and histone acetylation. Hence, kefir and kefir-derived isolated microorganisms and peptides increased the anti-inflammatory cytokines and decreased the pro-inflammatory cytokines responses, demonstrating its great anti-inflammatory potential. 

### 3.6. Antioxidant

Antioxidants are free-radical scavengers. They prevent damage produced by unstable molecules or free radicals which the body generates under stress and other environmental pressures. Kefir has strong antioxidant potentials, and it has been proven in both in vitro and in vivo models. A study by Yilmaz-Ersan et al. [130] reflected that kefir samples fermented using kefir grains exhibited better antioxidant potency, which was measured by the 2,2-diphenyl-1-picrylhydrazyl (DPPH) and 2,20-azino-di(3-ethylbenzthiazolin-sulfonate) ABTS assays, than kefir samples fermented by started cultures. The study implied that ewe milk kefir revealed an antioxidant effect in the ABTS assay. The isolated exopolysaccharide from Tibetan kefir grains during milk fermentation showed high antioxidant activities in in vitro and concentration-dependent protection of protein from oxidative injury [131]. On the other hand, Sabokbar et al. [132] discovered that the addition of kefir to apple juice increased the total phenolic content and antioxidant activities analyzed using diphenyl-2-picrylhydrazyl radical scavenging, reducing power, metal chelating effect, inhibition of linoleic acid autoxidation and inhibition of ascorbate autoxidation assays. Bensmira and Jiang [133] reported that kefir fermented in peanut milk demonstrated enhanced antioxidant effects versus peanut milk alone, suggesting the kefir grain’s fermentation impact on peanut milk’s efficacy. Likewise, kefir fermented with a mixture of cow and soy milk showed improved antioxidant activity compared to kefir fermented with cow’s milk alone, and the study found an increased level of phenolic compounds after the fermentation [134]. 

Kefir was also found to have significant antioxidant effects against reactive oxygen species. Ghoneum et al. [135] studied the protective activities of a novel kefir product (PFT) on 10-month-old oxidative stress-induced mice. This study showed that administration of PFT significantly increased antioxidant enzyme activities of superoxide dismutase, catalase, and glutathione peroxidase; decreased oxidative stress biomarkers nitric oxide, and malondialdehyde; reversed reductions in total antioxidant capacity, glutathione levels, and anti-hydroxyl radical content; inhibited liver enzyme levels of alanine aminotransferase (ALT) and aspartate aminotransferase (AST); significantly enhanced high-density lipoprotein levels; and significantly reduced total cholesterol, triglyceride, and low-density lipoprotein levels. Interestingly, the study found that administration of PFT reversed oxidative changes associated with ageing, hence normalizing the levels to young control mice in the brain, blood, and liver. Similarly, Radhouani et al. [136] evaluated the in vitro antioxidant properties of kefiran biopolymer. The study showed kefiran extract to possess the highest superoxide radical and reducing power scavenging activities. In addition, kefiran extract showed a great potency to scavenge nitric oxide radical. The study also demonstrated the potency of kefiran extracts on hASCs to improve its cellular function, without causing any cytotoxic responses. The study suggests a great potency of kefiran extract as a scavenger for reactive oxygen and nitrogen species, which could be an excellent candidate to promote tissue regeneration and repair. 

Sirirat and Jelena [114] investigated the antioxidant activity and bacterial inhibition between 24 and 48 h of Thai jasmine rice milk-kefir and cow milk-kefir. The rice milk-kefir showed higher bioactivity compared to the cow milk-kefir. The rice milk-kefir also resulted in a greater antioxidant activity measured using DPPH radical scavenging activity, hydroxyl radical scavenging activity, and lipid peroxidation assay, showing the potential of rice milk-kefir as a bacterial inhibition and oxidative damage mitigating agents. McCue et al. [137] used active probiotic cultures of kefir to study the phenolic antioxidant mobilization during the production of yoghurt from soymilk. The study demonstrated an increased content of the soluble phenolic along with kefir culture time, which also, in turn, increased its antioxidant activity. Another study by Alsayadi et al. [138] examined the antioxidant potency of water kefir. The study showed that water kefir scavenged the DPPH free radical from 9.88% to 63.17%, and inhibited the ascorbate oxidation by 6.08–25.57%, demonstrating its potential as an antioxidant agent. A study by Nurliyani et al. [139] investigated the properties of kefir fermented with a combination of black rice extract and goat milk and its effects on streptozotocin-nicotinamide (STZ-NA)-induced diabetic rats. The study demonstrated the antioxidant activity of kefir prepared from the combination of black rice extract and goat milk was higher compared to goat milk kefir only. The study also found that kefir fermented from goat milk and black rice extract could improve beta-cells regeneration which is similar to the glibenclamide, an antidiabetic agent. The study suggested that beta-cells regeneration was due to bioactive compounds in kefir which affected the beta-cells’ metabolism.

Liu et al. [140] examined the antioxidant and antimutagenic properties of kefir fermented in milk and soymilk. The study showed that milk-kefir and soymilk-kefir resulted in a significantly enhanced antimutagenic activity compared to the milk and soymilk controls. The milk-kefir and soymilk-kefir also showed a greater DPPH scavenging activity, decreased glutathione peroxidase activity, and inhibition effect upon linoleic acid peroxidation. The study also demonstrated that fermentation of kefir grains in milk and soymilk did not change the superoxide dismutase activity and ferrous ion chelating ability of the original materials. Satir and Guzel-Seydim [141] assessed the influence of kefir fermentation in Hair and Saanen goat milk on the bioactive substances. The study showed increased antioxidant activity in kefir fermented samples. Interestingly, the kefir grains fermented in goat milk resulted in higher total antioxidant activity and microbiota content. The phenolic contents in goat milk-kefir samples ranged between 726.08 and 1359.32 mg of gallic acid equivalents (GAE) L-1. The phenolic compounds in goat milk-kefir samples ranged between 0.36 and 5.09 mg 100 g^−1^ for catechin, and 0.77 and 4.21 mg 100 g^−1^ for gallic acid, showing significantly high bioactive substances in goat milk-kefir samples. A recent study by Koohian et al. [142] examined the radioprotective effects of kefir and ascorbic acid against radiation-induced DNA injury and genotoxicity in mice blood lymphocytes. The study showed that total comet score value in kefir and ascorbic acid groups were reduced 1.39- and 1.5-fold, respectively. The co-administration of kefir and ascorbic acid reduced DNA damage in lymphocyte blood cells. It was also revealed that the combination of kefir and ascorbic acid resulted in high antioxidant activities in both DPPH radical scavenging and ferric reducing antioxidant power assays, proving its antioxidant potential in protecting animal lymphocyte blood cells from radiation-induced DNA injury and genotoxicity. 

### 3.7. Hypocholesterolemic Effect

Kefir has high cholesterol-lowering properties, and it has been mostly validated in animal models. Yusuf et al. [143] have demonstrated that *Lentilactobacillus kefiri* strains and *Lacticaseibacillus rhamnosus* (basonym *Lactobacillus rhamnosus*) isolated from Indonesian kefir grain reduced the cholesterol from the media from 22.08% to 68.75%. However, the highest reduction was shown by *Lentilactobacillus kefiri* JK17. The study also manifested a significant decrease in the removal of cholesterol from the media in resting cells (14.58%–22.08%) and dead cells (7.89%–18.17%), indicating that the cholesterol-lowering effect can only occur when cells are metabolically active. A study by Liu et al. [144] showed that milk kefir and soy milk kefir administration to male golden Syrian hamsters fed with cholesterol-enriched and cholesterol-free diet resulted in reduced total cholesterol and serum triacylglycerol levels and improved atherogenic index, indicating that kefir administration altered the endogenous cholesterol metabolism. The study also found low level of cholesterol concentrations in the liver of hamsters treated with both milk and soy milk kefirs. Moreover, the secretion levels of fecal cholesterol and bile acid were also found to be significantly increased in both groups. The secretion of higher levels of cholesterol in fecal matter was most likely due to inhibition of cholesterol absorption in the small intestine due to assimilation and binding of cholesterol by microbes present in kefir. On the other hand, increased level of fecal bile acid is likely due to deconjugation of bile acid by the same microbes present in kefir.

Zheng et al. [145] showed that administration of *Lactobacillus acidophilus* LA15, *Lactiplantibacillus plantarum* (basonym *Lactobacillus plantarum*) B23, and *Lentilactobacillus kefiri* D17 decreased the total serum cholesterol, LDL cholesterol, and triglyceride levels in SD rats fed with a cholesterol-enriched diet. Another study by Wang et al. [146] demonstrated the hypocholesterolemic activity of *Lactiplantibacillus plantarum* (basonym *Lactobacillus plantarum*) MA2 isolated from kefir in male Sprague-Dawley rats fed with a cholesterol-enriched diet. The study found that the addition of this organism in the rats’ diet had significantly lowered the total serum cholesterol, triglycerides, LDL-cholesterol, liver cholesterol and triglycerides together with an increased level of fecal cholesterol secretion. Likewise, a study by Huang et al. [147] also showed that *Lactiplantibacillus plantarum* (basonym *Lactobacillus plantarum*) strains Lp09 and Lp45 supplemented in high cholesterol diet in SD rats had similar effects as the previous study. Another study by Huang et al. [148] has demonstrated that *Lactiplantibacillus plantarum* (basonym *Lactobacillus plantarum*) Lp27 supplementation in hypercholesteraemic SD rats was able to lower the total serum cholesterol, LDL-cholesterol, and triglycerides. A proposed that the mechanism is microbes present in kefir were responsible for decreasing the serum cholesterol by inhibiting the cholesterol absorption in the small intestine. It was reported that the Niemann–Pick C1-Like 1 (NPC1L1) gene that plays a crucial role in the cholesterol absorption mechanism was down-regulated in in vitro tests with Caco-2 cells and rats fed Lp27 organism [148]. 

## 4. Commercial Kefir Products 

Nowadays, consumers are showing great interest in functional foods as they become more aware of healthy eating for health and wellness. According to Guneser et al. [149] the global sales of functional foods and beverages associated with health benefits are expected to thrive by 2022. In fact, by 2020 the global sales for this market category worth were estimated to reach 377.8 billion US dollars [150]. It was recognized that functional beverages are the most active functional foods category exerting physiological effects on the body apart from providing basic nutrition [149,151]. Kefir is a nutritious alcoholic beverage that has been produced and consumed for centuries in central Asia and Eastern European countries. Due to the number of proclaimed health benefits of this product, there is an immense interest in other parts of the world including the United States, Germany, France, United Kingdom, Netherlands, Brazil, China, Japan, Turkey, Malaysia, Indonesia, Tibet, and North and South America.

Evidence of kefir’s global popularity is apparent through several commercial producers of kefir around the world. This includes Lifeway kefir (USA, UK, and Canada), Bionova (Italy), milk (Austria), Evolve Kefir (USA), Wallaby Organic (Australia), and CocoKefir (USA) [11]. Prado et al. [3], and Table 3 summarized the marketed kefir-based products provided by these companies with some of their general information. Lifeway is one of the largest kefir companies resulted from homemade kefir production by its founder that started in 1986. With over 15 different types of kefir products, Lifeway was able to boost its annual company revenues to over $120 million in 2017 and has expanded its distribution throughout the United States, Mexico, the UK, and Ireland as well as portions of Central and South America and the Caribbean. Although the kefir’s market in Malaysia is not widely known as in Europe countries, there are several growing kefir companies established in Malaysia including MyKefirWorld, MayKef Malaysian Kefir, and MooMoos Milk Kefir.

The growing popularity of kefir and kefir grains has prompted to the use of kefir starters in cheese production [152]. Magalhães et al. [153] demonstrated an innovative usage of kefir grains as a starter culture in the production of fermented cheese whey-based beverages. The study revealed a steady grain structure and dominant microbiota, including probiotic bacteria which unfold preservation of the properties of kefir when using whey as substrates. The fact that direct use of kefir grains is unrealizable due to transportation, storage, and cell concentration, had shifted into the usage of grain-free production (freeze-dried or thermally-dried kefir) at the commercial level. A similar study that used freeze-dried kefir as starter culture in the production of kefir-whey-cheese, revealed an acceleration of cheese ripening and resulted in enhanced sensory attributes [154]. Dimitrellou et al. [155] also investigated the use of thermally-dried immobilized kefir on casein as starter culture in dried whey cheese production. The study showed a significantly reduction of pathogens (*coliforms*, *enterobacteria* and *staphylococci*) which suggested repression of spoilage in cheeses produced using thermally-dried kefir starter cultures. In addition to a longer shelf life, superior aroma, taste, and texture were also found in cheese made using kefir inoculum [156,157].

The anti-microbial action of kefir grains has offered an added incentive for use as a bakers’ yeast in bread baking. Mantzourani et al. [158] showed that the use of kefir grains as a leavening agent in sourdough bread produces a product with longer shelf life than sourdough bread prepared with wild microflora, in terms of delay in manifestation of rope spoilage caused by *Bacillus* spp. The freshness of sourdough bread was also examined through the fragrant volatile composition during storage, which revealed that bread made with kefir sourdough manifested more complex profiles of volatiles with lower loss rates during storage than bread made with commercial sourdough [159]. Not only kefir grains preclude the need to use chemical preservatives (sodium, potassium, and calcium salts of propionic acid) in bread making, bread produced with kefir retained more moisture, had a good loaf volume and lower acidity (pH 4.9–5.5) that consumer like [12,159,160,161].

Kefiran, a potential polysaccharide produced from the kefir fermentation is stated as the most preferable EPS among others due to its water-soluble and biodegradable feature [162]. In fact, kefiran is a striking option over other EPS, including alginate, glucans, dextrin, xanthan, and levan due to its antitumor, antibacterial, antifungal, and immunomodulation activities that have been extensively studied [3,8,9,10,163]. Kefiran was successfully incorporated into various films providing a naturally derived packaging product resistant to contamination with added health-promoting features. The plasticizers, such as glycerol were employed to increase extensibility and water vapor permeability but decreased tensile strength [164,165,166]. Moreover, its viscoelastic and rheological properties of acid milk gels are broadly examined in the food industry as an emulsifier, stabilizer, and gelling agent [167,168,169,170,171]. Kefiran is, therefore, clearly a valuable component in biodegradable edible films that addressed both health and environmentally conscious consumers [12].

A recent study on health views towards kefir that correlated emotion and attitude using an emoji scale in Brazil revealed a remarkable positive acceptance among the consumers. The results significantly showed increased positive emojis attributed to emotional responses in liking and acquire intent to added kefir beverages after they had been enlightened of its health advantages [172]. Moreover, other alternative substrates such as fruits and molasses contribute to enhanced distinct sensory characteristics for adaptation of kefir product development and marketing strategies.

## 5. Future Prospect and Limitations

The wide array of possibilities for development of kefir-based products in the food and pharmaceutical industries has led to an in-depth look into kefir consumption over the past three decades. An overwhelming volume of advances in the existing knowledge has resulted in further exploration of possible kefir products and increased opportunities for providing health benefits to consumers.

In response to the growing and insistent demand for fewer synthetic preservatives among consumers, its promising anti-microbial activity as bio-preservatives were studied. The bacteriocins are a group of antimicrobial peptides that have been considered as one of the novel bio-preservatives to combat foodborne pathogens. A bacteriocin (F1) purified from *Lacticaseibacillus paracasei* subsp. *tolerans* (basonym *Lactobacillus paracasei* subsp. *tolerans*) FX-6 in Tibetan kefir, has shown potent anti-microbial activity against a broad spectrum of Gram-positive and Gram-negative bacteria and fungi [173]. In the later studies, the actual anti-microbial mechanisms of peptide F1 on *Escherichia coli* and *Staphylococcus aureus* were demonstrated necessary to improve its application in food preservation. The mechanisms involved were the ability to increase the outer and inner membrane permeability, disrupt bacterial cell membranes and bind to the genomic DNA in the cytoplasm, which collectively led to rapid cell death [96,174]. In another study, the extended shelf life and good quality of stored pork after 12 days confirmed the great promise of peptide F1 anti-microbial activity as bio-preservatives with clean-label status [175]. The kefir drink also was effective against *Aspergillus flavus* contamination on an arepa, while it retained the organoleptic characteristics of the traditional corn product [111]. The fact that the kefir products such as kefiran that inhibits pathogens, particularly in the GI and being resistant to hydrolytic enzymes has opened the door to kefir being developed into an adjuvant natural alternative antibiotic [14]. For instance, Blandón et al. [176] demonstrated that the exopolysaccharide, kefiran has not only enhanced the rheological properties of kefiran-alginate hybrid gel microspheres against harsh acid gastric conditions for oral delivery but also was able to show synergic antimicrobial activities between ciprofloxacin and kefiran against the GI pathogens. Additionally, its medical applications can also be applied through its ability in killing one of the most lethal enteric pathogens, *Clostridium difficile*, in which the artificial antibiotics can be reduced or even tapered off [177].

Remarkably, the administration of kefir beverages has recently been shown to modulate the composition of the host gut microbiota. Furthermore, there is burgeoning scientific evidence to suggest that imbalance of the intestinal microbiota is associated with immunopathologies, chronic diseases, as well as intestinal infections [109,178,179]. The environmental cause of the alteration in the gut microbiome is often attributed to antibiotic usage and poor dietary intakes. Interestingly, the use of probiotics has been demonstrated to be efficient of stimulating homeostasis of the intestinal microbiota, and has beneficial roles leading to potential control of gut microbiome-associated diseases (antibiotic-associated diarrhea (AAD), inflammatory bowel disease (IBD), Crohn’s disease (CD), colorectal cancer (CRC)) [180,181]. This opens possibilities for regular consumption of kefir to reduce the risk of gut dysbiosis, by changing and or improving the amounts and types of probiotics that live in the gut for the prevention of metabolic diseases [182,183].

A randomized trial in twelve subjects with metabolic syndrome whose diets were supplemented with 180 mL/day kefir showed the modulation of intestinal microbiota, with a significant rise in *Actinobacteria*, as well as variations in the genera of the phyla Bacteriodetes and Firmicutes [181]. The results observed intervenes with the metabolic factors, property of metabolic syndrome, recovery in fasting insulin and insulin resistance index (HOMA-IR), and a decline in pro-inflammatory cytokines, and systolic and diastolic pressure, proving the modulation of the human gut microbiota through the kefir consumption in patients with metabolic syndrome. Moreover, Bengoa et al. [184] evaluated the capacity of the EPS produced by two *L. paracasei* strains isolated from kefir grains, to be metabolized in vitro by the fecal microbiota. It was observed that both EPS produced CIDCA 8339 (EPS8339) and CIDCA 83124 (EPS83124) that led to changes in fecal microbiota with a significant increase in the production of propionic acid and butyric acid. The results revealed the exopolysaccharides of kefir as potential bioactive compounds for microbiota modulation with beneficial effects both at the gastrointestinal and extra-intestinal levels. Aside from the impact of kefir on the microbiome in diseases, its protective effects in healthy individuals also have been demonstrated [185]. For instance, Toscano et al. [186] demonstrated a randomized trial in 20 Italian healthy people whose diets were supplemented with *L. kefiri* for one month. The results showed significant reduction in several bacterial genera directly involved in the onset of pro-inflammatory response and gastrointestinal diseases, which concluded the ability of *L. kefiri* to modulate the gut microbiota composition. Hence, the compositions of kefir are both potentially exerted beneficial effects in healthy individuals as well as in some disease conditions due to its gut microbiota-altering role.

Furthermore, recently there has been an immense interest in the studies of altered gut microbiota in response to mental health upon kefir consumption. In fact, the term kefir derived from the Turkish word “keyif”, indicate a good feeling and pleasure after consumption suggested its potential gut-brain axis relationship. Murray et al. [187] studied the vital role of the gut microbiota in adolescent around the fact that the probiotics consumption can alter brain chemistry and behavior. In this context, the gut microbiota regulates stress and inflammatory responses with probiotics during puberty in CD1 mice on lipopolysaccharide (LPS)-induced immune responses. The presence of the probiotic effect could be considered to be the ability to produce anxiolytic effects and decreases stress-induced vulnerabilities during pubertal neurodevelopment. A recent study on the interaction of microorganisms present in kefir grains with the microorganisms present in the GI of zebrafish, demonstrated that kefir associated with soybean germ (KSG) and fermentation solution of kefir (FSK) exhibit anxiolytic and antidepressant effects [188]. This observation suggested that the essence of the kefir contribute to the modulation of the host’s intestinal microbiota, thus allowing the positive immunomodulation and resulting in the improvement of mental health.

Kefir has been attributed as a homemade probiotic drink with low production cost and also as a natural folk remedy. However, some studies suggested that artisanal kefir manufacturing may resulted in kefir contamination associated with poor hygiene practices and, thus, pose possible health risks for consumers. Angelidis et al. [115] investigated the *Staphylococcus aureus* growth and enterotoxin production during domestic kefir fermentation affiliated with the initial level of *S. aureus* contamination due to failure in sticking to good hygiene practices. The richness of kefir microbiota and metabolites that contribute to its probiotic properties is undoubted. However, the industrial production of kefir on a larger scale is still scarce that resulted in the limitation for methodological standardization. As described previously, the need for a standardized kefir manufacturing condition is crucial to fully exploit the physiological benefits of kefir. It is worth mentioning that the different manufacturing conditions of kefir (agitation; the inoculum concentration; as well as the fermentation time and temperature) may alter the original characteristics of the microbial composition, hence affecting their health-giving properties [11]. Thus, the advances of various -omic technologies, including proteomics, transcriptomics, genomics, metabolomics, lipidomics, and epigenomics, may provide a greater insight into the isolation and utilization of the exact kefir microbes and/or metabolites responsible for measurable health benefits [13,189]. 

## Figures and Tables

**Table 1:** Anti-cancer activities of kefir and its cell-free extracts.

Kefir	Biological Activity	Reference
Milk kefir and soy milk kefir	Anti-inflammatory effect on murine sarcoma (in vivo)	Liu et al., 2002 [52]
Kefram–Kefir aqueous extract	Anti-oxidant and apoptosis effect on human melanoma cell line HMV-1/SK-MEL (in vitro)	Nagira et al., 2002 [53]
Kefir and kefir cell-free fraction (KF)	Anti-inflammatory effect on murine breast cancer (in vivo)	de Moreno de LeBlanc et al., 2006; De Moreno De LeBlanc et al., 2007 [54,55]
Cell-free fraction of kefir	Anti-proliferative effect on human mammary cancer cell line MCF-7 (in vitro)	C. Chen et al., 2007 [56]
Cell-free fraction of kefir	Anti-proliferative and apoptosis effect on human T-cell leukemia cell line HuT-102 (HTLV-1 negative/HTLV-1 positive) (in vitro)	Rizk et al., 2009; 2013 [57,58]
Cell-free fraction of kefir	Anti-proliferative and apoptosis effect on HTLV-1-negative malignant T-lymphocytes (in vitro)	Maalouf et al., 2011 [59]
Cell-free fraction of Tibetan kefir	Anti-proliferative effect on human gastric cancer cell line SGC7901 (in vitro)	Gao et al., 2013 [60]
*Lentilactobacillus kefiri*	Apoptosis of human myeloid leukemia cell line HL60/AR (in vitro)	Ghoneum & Gimzewski, 2014 [61]
Cell-free fraction of kefir	Anti-proliferative and apoptosis effect on colorectal cancer cell line Caco-2/HT-29 (in vitro)	Khoury et al., 2014 [62]
*Lentilactobacillus kefiri*	Apoptosis in human gastric cancer cell line (AGS)	Ghoneum & Felo, 2015 [63]
Cell-free fraction of kefir	Anti-proliferative and apoptosis effect on human acute erythroleukemia cell line KG-1 (in vitro)	Jalali et al., 2016 [64]
Kefir, Exopolysaccharides(EPS), Alkaline Kefir (AK), Alkaline EPS (AEPS)	Apoptosis in human sarcoma cells (in vitro)	Almostafa Alsha’ar et al., 2017 [65]
Exopolysaccharide from *Lentilactobacillus kefiri* MSR101	Anti-proliferative and apoptosis effect on human colon cancer cell line HT-29 (in vitro)	Riaz Rajoka et al., 2019 [10]
Milk kefir		
Cell-free fraction of kefir	Adjuvant anti-cancer effects on multidrug-resistant human colorectal cancer cells (HT-29)	Kim et al., 2021 [66]

**Table 2:** List of pathogenic organisms that kefir or kefir-associated organisms have demonstrated antimicrobial effects against.

Microbial Species	Reference
**Bacteria**	
*Bacillus cereus*	Anselmo et al., 2010; Carasi et al., 2014; Kakisu et al., 2007; Medrano et al., 2008; Ulusoy et al., 2007 [80,81,82,83,84]
*Bacillus subtilis*	Chifiriuc et al., 2011 [85]
*Bacillus thuringiensis*	Miao et al., 2014 [86]
*Clostridium difficile*	Bolla et al., 2013; Rea et al., 2007 [87,88]
*Clostridium perfringens*	Anselmo et al., 2010 [80]
*Enterococcus faecalis*	Chifiriuc et al., 2011; Zanirati et al., 2015 [85,89]
*Escherichia coli*	Chifiriuc et al., 2011; Ebner et al., 2015; Garrote et al., 2000; Golowczyc et al., 2008; Gulmez & Guven, 2003; E. Kakisu et al., 2013; A. M. O. Leite et al., 2015; Meng et al., 2017; Miao et al., 2014; Miao, Liu, et al., 2016; Morgan et al., 2000; Rodrigues et al., 2005; Santos et al., 2003; Silva et al., 2009; Suriasih, 2011; Ulusoy et al., 2007; Yüksekdag et al., 2004; Zanirati et al., 2015 [43,84,85,86,89,90,91,92,93,94,95,96,97,98,99,100,101]
*Helicobacter pylori*	Oh et al., 2002; Zubillaga et al., 2001 [102,103]
*Klebsiella pneumoniae*	Garrote et al., 2000; Yüksekdag et al., 2004 [90,101]
*Listeria innocua*	Morgan et al., 2000; Powell et al., 2007 [97,104]
*Listeria monocytogenes*	Gulmez & Guven, 2003; Jeong et al., 2017; A. M. O. Leite et al., 2015; Likotrafiti et al., 2015; Rodrigues et al., 2005; Santos et al., 2003; Ulusoy et al., 2007; Zanirati et al., 2015 [9,84,89,92,94,98,99,105]
*Pseudomonas aeruginosa*	Carasi et al., 2014; Huseini et al., 2012; Rahimzadeh et al., 2011; Rodrigues et al., 2005; Yüksekdag et al., 2004; Zanirati et al., 2015 [81,89,98,101,106]
*Salmonella enterica*	Golowczyc et al., 2008; A. M. O. Leite et al., 2015; Miao et al., 2014 [86,91,94]
*Salmonella* Enteriditis	Czamanski et al., 2004; R. J. Anselmo et al., 2001; Carasi et al., 2014; Chifiriuc et al., 2011; M. A. Golowczyc et al., 2007; Jeong et al., 2017; Santos et al., 2003; Ulusoy et al., 2007 [9,81,84,85,99,107,108]
*Salmonella* Gallinarum	Golowczyc et al., 2008 [91]
*Salmonella* Typhimurium	Marina A. Golowczyc et al., 2008; Meng et al., 2017; Rodrigues et al., 2005; Santos et al., 2003; Silva et al., 2009; Suriasih, 2011; Zanirati et al., 2015 [89,91,95,98,99,100]
*Shigella dysenteriae*	Miao et al., 2014 [86]
*Shigella flexneri*	P. A. Bolla et al., 2016; Santos et al., 2003 [99,109]
*Shigella sonnei*	Golowczyc et al., 2008; Silva et al., 2009 [91,100]
*Staphylococcus aureus*	Carasi et al., 2014; A. M. O. Leite et al., 2015; Miao, Zhou, et al., 2016; Rodrigues et al., 2005; Silva et al., 2009; Ulusoy et al., 2007; Yüksekdag et al., 2004; Zanirati et al., 2015 [81,84,89,94,96,98,100,101]
*Staphylococcus salivarius*	Rodrigues et al., 2005 [98]
*Streptococcus faecalis*	Ismaiel et al., 2011 [110]
*Streptococcus pyogenes*	Rodrigues et al., 2005 [98]
**Fungi**	
*Aspergillus flavus*	Gamba et al., 2016; Miao et al., 2014 [86,111]
*Aspergillus niger*	Miao et al., 2014 [86]
*Aspergillus ochraceus*	Caro Vélez & León Peláez, 2014 [112]
*Candida albicans*	Rodrigues et al., 2005; Silva et al., 2009 [98,100]
*Fusarium graminearum*	Ismaiel et al., 2011 [110]
*Penicillium glaucum*	Miao et al., 2014 [86]
*Rhizopus nigricans*	Miao et al., 2014 [86]
*Staphylococcus epidermidis*	Topuz et al., 2008 [113]
*Yersinia enterocolitica*	Gulmez & Guven, 2003; Santos et al., 2003 [92,99]

**Table 3:** Marketed kefir-based products and their information.

Companies	Product	General Information
Bionova	Milk kefir (Natural, vanilla, blueberry, barley, ginger and strawberry)	Natural source of calcium, gluten-free and produced from high-quality milk.
	Kefir water (Natural, wellness, red fruits, relax, antioxidant and energy)	Natural source of calcium and gluten-free.
	Probioflora kefir	Food supplement contains freeze-dried granules of Kefir and selected live lactic ferments.
	Actiplus kefir	Natural source of calcium and gluten-free packed with billions of live and active lactic ferments.
milk	Kefir Plus (Tropical fruits, blueberry, royal jelly)	Lactose-free fermented cow’s milk with live lactic ferments specific to kefir, a natural source of calcium and vitamin.
	Kefir to Drink (Natural white, orange ginger and cardamom, multi-fruits, mango turmeric, pomegranate and raspberry,	Lactose-free fermented cow’s milk with live lactic ferments specific to kefir, a natural source of calcium and vitamin.
	Kefir to Drink Bio (Natural white)	Lactose-free fermented cow’s milk produced from organic farming with live lactic ferments specific to kefir, a natural source of calcium and vitamin.
	Creamy Kefir (White, strawberry and wild strawberries, blackberry, plum and cereals, pomegranate and chia, oats and nuts)	Lactose-free fermented cow’s milk kefir with milk cream, with live lactic ferments specific to kefir, a natural source of calcium and vitamin.
	Kefir Mix White (With wholemeal cornflakes and red fruits, with pumpkin, sunflower and cranberry seeds, with crunchy hazelnut, with berries).	Lactose-free fermented cow’s milk kefir with milk cream mixtures, with live lactic ferments specific to kefir, a natural source of calcium and vitamin.
